# The salivary gland as a target for enhancing immunization response

**DOI:** 10.1186/s40794-017-0047-z

**Published:** 2017-02-17

**Authors:** Todd A. Ponzio, John W. Sanders

**Affiliations:** 1 Naval Medical Research Center, 503 Robert Grant Ave., Silver Spring, MD 20910 USA; 2 Wake Forest University School of Medicine, Winston-Salem, USA; 3 Wake Forest University School of Medicine, Medical Center Blvd., Winston-Salem, NC 27157 USA; 4 Hefner Veterans Affairs Medical Center, Salisbury, NC UK

**Keywords:** Vaccine, Immunization, Intramuscular, Salivary gland, Mucosal immunity, Enteric vaccine

## Abstract

**Background:**

An organism’s immune response to a vaccine is dependent on a number of factors, including the site of immunization. While muscle is the most common site for vaccine administration, other sites, including the salivary gland, are poised to confer stronger and broader immunoprotection.

**Findings:**

Studies exploring the salivary gland as an immunization site have involved protein antigens, as well as live pathogens and DNA vaccines. While intraductal instillation of protein antigens into the salivary gland may result in a relatively transient increase in antibody production, DNA or attenuated pathogen vaccination appear to confer a lasting widespread mucosal immune response that includes robust salivary and enteric IgA, as well as high levels of circulating IgG. Furthermore, vaginal and lung antibodies are also seen. For enteric pathogens, a common class of pathogen encountered by travelers, this type of immune response provides for a level of redundant protection against foreign microbes with mucosal targets.

**Conclusion:**

The strength of immune response conferred by salivary gland vaccination is generally stronger than that seen in response to the same vaccine at a comparison site. For example, where other routes fail, immunization of the salivary gland has been shown to confer protection in lethal challenge models of infectious pathogens. A host of vaccines currently under development suffer from immunogenicity challenges, adding to the widespread interest and search for novel routes and adjuvants. With its capability to facilitate a strong and broad immune response, the salivary gland warrants consideration as an immunization site, especially for vaccines with immunogenicity challenges, as well as vaccines that would benefit from combined systemic and mucosal immunity.

## Background

Vaccine delivery leading to systemic antibody-mediated immunity is usually administered through intramuscular injection (IM). Although the resulting humoral immune response forms the basis for protection conferred by nearly all vaccines delivered today, immune responses to the vaccines for bacille Calmette-Guerin (tuberculosis) and zoster trigger a strong cell-mediated immunity component [1]. Most pathogens, however, enter the body through mucosal tissues, including the aerodigestive and urogenital tracts, the eye conjunctiva and external-interfacing exocrine glands. Given the constant interaction with the environment, the mucosal immune system has evolved efficient mechanisms to maintain tolerance toward self-antigens as non-pathogenic environmental antigens, as well as mount active immune responses against salient pathogens—features which make mucosal interfaces particularly attractive sites for vaccine delivery [2].

While most vaccines continue to be administered parenterally, a number of mucosal vaccines are commercially available. The two routes of administration for the currently licensed mucosal vaccines are oral and nasal, and examples of orally-administered vaccines include vaccines for rotavirus, poliovirus, and cholera [3]. Mucosal vaccines for various influenza viruses are delivered intranasally, and a recent exploratory clinical trial comparing a live attenuated influenza vaccine (FluMist®) administered via the intranasal or sublingual route reported haemagglutination inhibition titers of 440 (*n* = 20) following intranasal delivery, and 380 (*n* = 20) following sublingual delivery [4]. The administration site also affects the amount of compartmentalization of the resulting mucosal immune response, with the sublingual route leading to broader immune responses as compared to oral and nasal administration [5]. Anatomically related to the sublingual route, another lesser-explored mucosal vaccination route includes intraductal instillation of the salivary gland.

There are three major bilateral salivary glands, the parotid, submandibular, and sublingual (collectively referred to as the SG). They are encapsulated exocrine glands responsible for the manufacturing of saliva, a complex fluid that includes significant amounts of peptides, proteins, and carbohydrates. The SG is also an immune organ, constantly coming in contact with external pathogens, and may be an optimal immunization site for certain vaccines. In addition to containing dendritic cells, a type of antigen presenting cell, the SG also appears to be a site of B cell activation and isotype switching, along with induction of antigen-specific cytotoxic T lymphocytes [6, 7].

### Whole-cell killed pathogen immunization of the salivary gland

Antibodies have long known to be present in saliva, though they have largely been viewed as potential by-product surrogate markers of exposure to various infectious diseases [8–10]. Varying consistency across readings and individuals has made the use of saliva as a diagnostic a challenge. However, IgA from targeted SG secretions, rather than whole saliva, has been shown to serve as a high-sensitivity readout for enteric infections, including enterotoxigenic *Escherichia coli* [11]. This will likely become a useful proxy in field trials for vaccines targeting enteric pathogens given that collecting and assaying targeted saliva is less resource-intensive than intestinal lavages or fecal samples.

While immunizations at various sites can result in measurable changes in salivary antibodies, both serum and salivary antibodies have been reported to result from repeated intraductal instillation of either protein or formalin-fixed pathogen into the SG. One early study found a strong IgA response following the injection of bovine serum albumin (BSA) into surgically-exposed SG [12]. However, the surgical procedure itself induces a natural immune and inflammatory response, and injections via needles traverse the connective tissue sheathing of the gland. A more direct and gentle route of applying a protein antigen was subsequently explored by an Israeli group.

With an aim toward gaining a better understanding of inflammatory processes in the parotid gland, the group assessed locally-secreted antibodies in response to antigen instillation of the parotid gland. Specifically, the group investigated both salivary and systemic antibodies resulting from intraductal instillation of BSA [13, 14]. The BSA was administered repeatedly a total of eight times, and serum and saliva samples were assayed for hemagglutinating anti-BSA antibodies. While they did not evaluate the relative changes in IgAs and IgGs, an increase in overall antibody titers of the saliva was seen. However, they reported only approximately one third of the animals had systemic hemagglutinating anti-BSA antibodies, a result that may relate to the use of BSA as the immunogen, which is a component in rodent food pellets, and therefore a potential immune toleragen [15, 16]. The ability for the SG to mount an immune response to protein or protein subunit antigens continues to remain an open question.

Around the same time as the Israeli group was exploring correlates of antigen-induced parotiditis, there was broad scientific effort in trying to develop a vaccine for dental caries, and specifically against the main offender, *Streptococcus mutans*. Formalin-killed *S. mutans* was being injected by various routes by different groups in an attempt to vaccinate various animal models from dental caries, including intravenously and subcutaneously in the mouth [17]. One group thought to investigate repeat intraductal instillation of non-human primate parotid gland. Following multiple oral subcutaneous immunization, the group then instilled formalized *S. mutans* bilaterally into the parotid glands of non-human primates [18]. While the subcutaneous immunization (total of 10 repeat administrations) led to modest increases in circulating IgM and IgG, the same subcutaneous immunizations did not change salivary IgA. However, intraductal instillation significantly increased both systemic IgG as well as salivary IgA. Buoyed by the results, the same group followed up with a study to assess efficacy (as measured by colonization), choosing to focus on immunizing only via intraductal instillation [19]. Both the number of infected surfaces, as well as the number of *S. mutans* organisms in a particular plaque were reduced in the immunized group.

A study comparing multiple subcutaneous immunizations in the vicinity of the salivary glands with multiple bilateral immunizations via intraductal instillation found the subcutaneous route induced only a serum response while intraductal instillation resulted in both serum and salivary IgA responses [20]. This finding was then later corroborated in a study instilling formalized S. *mutans* three times in a single parotid [21]. While these results were indeed promising, elevated specific antibody titers did not last long; and a commercial vaccine that required routine boosting would be a challenge from a commercial perspective. Live vaccines can confer more enduring protection, but due to the safety concerns related to live vaccines, DNA vaccines present themselves as promising alternatives, provided that any given DNA vaccine is sufficiently immunogenic [22]. Immunogenicity, however, is one of the more common challenges associated with DNA vaccines, and in this regard, the SG may have tempting appeal.

### Genetic vaccination of the salivary gland

The first study to target the SG as a potential immunization site using plasmid DNA (pDNA) employed an expression system coding for *Porphyromonas gingivalis* fimbriae [23]. Strong humoral and cell-mediated immune responses were seen in BALB/c mice following transcutaneous targeted salivary gland (TTSG) immunization (see Table 1). Though similar systemic titers of IgG were seen in response to IM or TTSG immunization, only TTSG elicited strong levels of salivary IgA. A more direct route of delivering a genetic vaccine to the SG was subsequently explored by a different group using a plasmid coding for the influenza NP protein. In this pilot study, pDNA complexed with the cationic lipid reagent adjuvants GAP-DLRIE/DOPE or Vaxfectin® was instilled intraductally into the SG in rats [24]. While the authors were unable to detect changes in salivary IgA, significant changes in systemic IgG were observed.

**Table 1:** A summary of various experiments using the Salivary Gland as an immunization site

Antigen	IgA, saliva	IgG, systemic	Reference	Model	Notes
Formalized *S. mutans*	++++	++++	[18]	NHP	SGid > SC; Greater IgA and IgG seen with SGid imzn than with SC imzn.
Formalized *S. mutans*	+++	+++	[21]	NHP	Greater IgA seen with SGid imzn than with SC imzn.
Formalized *S. mutans*	++++	++++	[19]	NHP	SGid imzn resulted in semi-protection against a *S. mutans* challenge.
Formalized *S. mutans*	++++	+++	[20]	NHP	SGid immunization was strain specific.
Bovine serum albumin	++^a^	++^a^	[13]	Rats	SGid; Moderate salivary and systemic antibody responses reported
Bovine serum albumin	++^a^	+^a^	[14]	Rats	Multiple SGid BSA imzns are followed by systemic antibodies.
MCMV, tcMCMV	n.a.	++++	[33]	Mice	SGig imzn resulted in protection from viremia; IP imzn did not.
MCMV, tcMCMV	+++	+++	[7]	Mice	SGig > IP; Greater IgA and IgG seen with SGig imzn than with IP imzn.
Adenovirus	+++	++++	[15]	Rats	SGid; Repeated pre-exposure to inactivated adenovirus induced immune tolerance.
tcMCMV	+++	++++	[34]	Mice	SGid imzn resulted in protection from a lethal challenge.
pDNA: fimbriae from *P.* *ginigivalis*	++++	+++	[23]	Mice	TSG > IM; Greater IgA seen with TSG imzn, than with IM imzn. TSG and IM imzns resulted in equal IgG.
pDNA: influenza NP protein	n.c.	+++	[24]	Rats	SGid; Included testing of two adjuvants.
pDNA: NP protein, hGH, gp120, anthrax PA	++++	++++	[25]	Rats	SGid > IM; Greater IgA and IgG seen with SGid imzn than with IM imzn. SG imzn protected from a lethal challenge; IM imzn did not; distal mucosal response seen.
pDNA: gp120	+++	+++	[27]	Rats, dogs	SGid > IM (ASCs from Peyer’s patches); distal mucosal IgA response seen in lungs.

*Abbreviations*: *SGid* intraductal instillation of the salivary gland, *SGig* direct injection into the gland through a small incision, *TSG* targeted injection of the salivary gland, *SC* subcutaneous injection, *IM* intramuscular injection, *IP* intraperitoneal injection, *imzn* immunization, *ASC* antibody secreting cell, *NHP* non-human primate, *MCMV* murine cytomegalovirus, *tcMCMV* tissue-cultured MCMV (i.e., attenuated MCMV), *anthrax PA* anthrax protective antigen, *hGH* human growth hormone, *gp120* HIV gp120, *pDNA* plasmid DNA, *n.c*. no change, *n.a*. not addressed. ^a^Antibody response confounded by immunogen being present in animal chow, possibly inducing immune tolerance; see [15, 16]. Table compiled by searching the PubMed database on “instillation duct antibodies” or “intraductal instillation antibodies,” analyzing salient peer-reviewed reports and their references, then subjecting those reports to a citation tracking database (Web of Science®) and analyzing the resulting reports, which were also run through a citation tracking database, with the salient reports evaluated as well

A more extensive study, also done in rats, involved the bilateral instillation of 88 μg of pDNA coding for either of three antigens, human growth hormone (hGH), HIV envelope protein gp120, or protective antigen (PA) from *Bacillus anthracis* (i.e., anthrax) [25]. As compared to IM and sublingual vaccination, higher titers of systemic IgG (and IgA) were seen in response to SG vaccination of plasmid encoding for hGH. Higher titers in response to SG, rather than IM, vaccination were observed for the gp120 genetic vaccine as well, and a memory response was observed. Circulating antibodies titers in response to SG and subcutaneous vaccination were more than ten-fold higher than those seen in response to IM vaccination with the PA gene; and the higher titers (but not the lower titers seen following IM vaccination) provided protection from a subsequent lethal anthrax challenge. Of particular interest to those involved in the development of novel mucosal vaccines were the observations of widespread mucosal antibody responses and significant increases in salivary and fecal IgA, as well as lung and vaginal IgG following SG vaccination with gp120 combined with a Zn/lipid adjuvant. Antibody-secreting cells (ASCs) isolated from Peyer’s patches following SG vaccination were approximately four times higher than those seen following IM vaccination. As antibody titer can predict efficacy, even for cell-mediated immunity, these results suggest it would be prudent to include the SG as a possible vaccination site [26].

Lastly, in an effort to explore the scalability of intraductal instillation of a DNA vaccine to larger animals, the method was extended to beagles in a follow-up study [27]. Solutions containing pDNA in the presence of Zn (with or without lipid) were instilled into the SG of the rats and beagles. The rats and dogs received bilateral instillations of 88 μg and 2.5 mg of pDNA, respectively, and the resulting antibody titers following SG vaccination were similarly high, registering at above 10,000. Addition of Zn improved protein expression, and a distal IgA response was also measured in the lungs. ASCs from Peyer’s patches were evaluated in this study as well, and SG immunization was seen to result in approximately 3–4 times as many ASCs as compared to IM immunization. Furthermore, there have been many described improvements related to the vector design of DNA vaccines, and optimizing future plasmids for instillation would only further enhance effectiveness [28]. Collectively, the data suggest the SG may warrant inclusion as a test vaccination route for mucosal vaccines under development, especially DNA vaccines that would benefit from increased immunogenicity.

### Live pathogen vaccination of the salivary gland

Though the SG is a well-established effector site for antibody-secreting B cells, induction of B cells is generally more restricted to specific sites, such as mucosa-associated lymphoid tissue of the ileum [29]. However, infiltrating leukocytes in other tissues (including SG) can form ectopic lymphoid-like structures, including organized aggregates that promote antigen-specific adaptive immune responses to include the proliferation of B cells and the production of antigen-specific antibodies [30]. In response to an immune challenge, the SG can form ectopic lymphoid-like structures that serve as inductive sites and which express activation-induced cytidine deaminase (AID), an enzyme required for somatic hypermutation and class switching [7]. This understanding helps explain what happens when mechanisms get misaligned, as in Sjogren’s syndrome, as well as the opportune results from studies instilling live pathogens [31].

Vaccination using live pathogens has long been demonstrated to elicit broad and lasting immune responses. Indeed, the first vaccines administered were all live [32]. In an effort to explore distal immune responses following SG inoculation, one study investigated tissue viral titers, tissue pathology, and antibody responses. The inoculum consisted of either murine cytomegalovirus (MCMV) or the less virulent tissue-culture-derived MCMV (tcMCMV) delivered through various routes, including injecting into the salivary gland through a small incision (intraglandular) [33]. While systemic IgG and IgM observed in response to intraglandular and intraperitoneal inoculation were similar, of all the routes tested (intraperitoneal, intranasal, periglandular and intraglandular), only intraglandular SG inoculation of tcMCMV was able to prevent splenic necrosis and hepatitis while limiting SG viral titers. Delivering tcMCMV into the SG was also shown to correlate with maintaining SG leukocyte infiltrates.

A subsequent comprehensive study by the same group employed the gentler route of intraductal instillation. Here, instilling either a replication-deficient recombinant adenovirus expressing individual MCMV genes or tcMCMV resulted in protection against a lethal systemic challenge with MCMV [34]. Due to its broad tropism and relative safety, the adenovirus is a common vector used to deliver vaccine antigens and other transgenes, and its potent immune reactions in the SG can be tolarized through pre-exposure [35]. Using a recombinant replication-deficient adenovirus to deliver MSMV genes resulted in strong systemic neutralizing MCMV-specific IgG in the serum [34]. Additionally, immunization with the adenovirus induced mucosal neutralizing MCMV-specific IgA at the primary immunization site (saliva), as well as distal mucosal sites (feces and vagina). This observation further corroborated and extended the findings by Tucker et al 2003, that SG vaccination with pDNA results in distal mucosal immune responses. Lastly, these immune responses provided vital protection in a lethal MCMV challenge. In light of the strong mucosal and systemic protective responses, the findings support the further testing of the salivary gland as a potentially attractive platform for active vaccine delivery.

## Conclusion

Here we have attempted to provide an overview of the SG as an immunization site, and synthesize a number of studies suggesting it be included as a selected immunization site in the development of novel vaccines, especially vaccines that do not elicit strong immune responses. Table 1 provides a qualitative analysis of salivary and systemic antibody responses following immunization of the salivary gland in several animal models. Antibody responses were measured by a host of methods, including hemagglutinating antibody titers, indirect immunofluorescent staining of the immunogen, ELISA optical density, and percent specific antibody values measured by radioimmunoassay, and a general qualitative magnitude of systemic IgG and salivary IgA response is provided.

As shown in the Table, various groups have demonstrated the SG to be capable of eliciting robust widespread mucosal and systemic protective responses in following antigen presentation, routinely resulting in antibody titers higher than those seen with other immunization targets. It is notable that studies have generally instilled the particular immunogen or vector (e.g. formalized S. mutans, BSA, virus, or plasmid DNA) in the absence of an adjuvant, yet still observed significant immune responses. Indeed, while complete Freund’s adjuvant has been used in studies employing subcutaneous immunizations delivered in the vicinity of the salivary glands (reviewed in [17]), very few studies using intraductal instillation have included potential immune adjuvants in the infusate (cationic lipid transfection reagents Vaxfectin® and GAP-DLRIE/DOPE in rats, or azo dye Evans blue in dogs) [24, 27]. Studies have generally instilled the test vaccine in a saline vehicle, achieving notable immune responses in the absence of an adjuvant. Given that vaccine efficacy is typically linked to antibody titer concentration, achieving high titers would be a common goal. A host of groups are exploring novel adjuvants to enhance immunogencitiy, the needs of which may be diminished if the vaccination site were able to facilitate a strong enough immune response.

While intraductal instillation into the SG is not an obvious route for vaccination, the potential benefits seen by a robust immune response would warrant the additional research effort. The practicality of mass immunization via the SG may be logistically challenging. However, infusion of the gland is a relatively simple procedure that can be done in an outpatient setting, such as during a routine dental check-up. While intraductal instillation is quick and painless, requiring neither needles nor anesthesia, most health-care workers have only modest familiarity with the procedure; and so training and instrumentation advances that simplify vaccine administration would significantly increase the amenability of the SG as a preferred site.

Additionally, saliva contains a number of carbohydrates, peptides, and enzymes, and this complex mixture may require novel formulation approaches to maximize vaccine efficiency and bring an SG-based vaccine into practical application. While the infusate vehicle in most SG immunization studies has been either water or a simple saline solution, the inclusion of mucoadhesives, protease, or nuclease inhibitors could further increase immunization efficiency. For example, adding zinc to the ductal infusate has been reported to result in a 20-fold increase in transgene expression, which would be important for DNA-based vaccines [36]. Nevertheless, the studies described above demonstrate striking capabilities of the SG as an immunization site. There are currently a host of antigen-based mucosal vaccines under development using live-attenuated pathogens, as well as DNA-based, carbohydrate, and protein antigens. While further studies are certainly needed to build upon the current literature discussed here, and specifically to compare SG intraductal instillation with other administration techniques into other tissues, the SG presents as an attractive route of administration.

## Abbreviations

AID: Activation-induced cytidine deaminase
ASC: Antibody secreting cell
BSA: Bovine serum albumin
DNA: Deoxyribonucleic acid
gp120: HIV gp120
hGH: Human growth hormone
IgA: Immunoglobulin G
IgG: Immunoglobulin A
IM: Intramuscular injection
imzn: Immunization
IP: Intraperitoneal injection
MCMV: Murine cytomegalovirus
n.a.: Not addressed
n.c.: No change
NHP: Non-human primate
NP: Nucleoprotein
PA: Protective antigen
pDNA: Plasmid DNA
SC: Subcutaneous injection
SG: Salivary gland
tcMCMV: Tissue-culture-derived MCMV
TTSG: Transcutaneous targeted salivary gland
Zn: Zinc
